# Classification of tomato seedling chilling injury based on chlorophyll fluorescence imaging and DBO-BiLSTM

**DOI:** 10.3389/fpls.2024.1409200

**Published:** 2024-09-09

**Authors:** Zhenfen Dong, Jing Zhao, Wenwen Ji, Wei Wei, Yuheng Men

**Affiliations:** ^1^ School of Arts and Sciences, Suqian University, Suqian, China; ^2^ School of Physics and Electronic Engineering, Yancheng Teachers University, Yancheng, China

**Keywords:** tomato, chilling injury, classification, chlorophyll fluorescence imaging, DBO-BiLSTM

## Abstract

**Introduction:**

Tomatoes are sensitive to low temperatures during their growth process, and low temperatures are one of the main environmental limitations affecting plant growth and development in Northeast China. Chlorophyll fluorescence imaging technology is a powerful tool for evaluating the efficiency of plant photosynthesis, which can detect and reflect the effects that plants are subjected to during the low temperature stress stage, including early chilling injury.

**Methods:**

This article primarily utilizes the chlorophyll fluorescence image set of tomato seedlings, applying the dung beetle optimization (DBO) algorithm to enhance the deep learning bidirectional long short term memory (BiLSTM) model, thereby improving the accuracy of classification prediction for chilling injury in tomatoes. Firstly, the proportion of tomato chilling injury areas in chlorophyll fluorescence images was calculated using a threshold segmentation algorithm to classify tomato cold damage into four categories. Then, the features of each type of cold damage image were filtered using SRCC to extract the data with the highest correlation with cold damage. These data served as the training and testing sample set for the BiLSTM model. Finally, DBO algorithm was applied to enhance the deep learning BiLSTM model, and the DBO-BiLSTM model was proposed to improve the prediction performance of tomato seedling category labels.

**Results:**

The results showed that the DBO-BiLSTM model optimized by DBO achieved an accuracy, precision, recall, and F1 score with an average of over 95%.

**Discussion:**

Compared to the original BiLSTM model, these evaluation parameters improved by 9.09%, 7.02%, 9.16%, and 8.68%, respectively. When compared to the commonly used SVM classification model, the evaluation parameters showed an increase of 6.35%, 7.33%, 6.33%, and 6.5%, respectively. This study was expected to detect early chilling injury through chlorophyll fluorescence imaging, achieve automatic classification and labeling of cold damage data, and lay a research foundation for in-depth research on the cold damage resistance of plants themselves and exploring the application of deep learning classification methods in precision agriculture.

## 1 Introduction

The greenhouse refers to a building that can control or partially control the growth environment of plants, and it has facilities such as cold protection, heating, and light transmission, and is mainly used in seasons that are not suitable for plant growth (Hanan, 2017), such as low temperature seasons. The severely cold regions of Northeast have a colder climate and large latitude span in China, and in winter and spring, outdoor temperatures may reach minus 40 degrees Celsius (Shao and Jin, 2020). Even if heating equipment is used to increase the temperature inside the greenhouse, it is difficult to always maintain the temperature at which plants adapt to their growth. Especially at night, the temperature will suddenly drop, making plants vulnerable to low temperature stress damage. Therefore, low temperature is one of the main environmental limiting factors affecting plant growth and development in Northeast China. Tomatoes (Lycopersicon esculentum) are one of the most important vegetables grown worldwide, and are warm loving plants that are sensitive to cold during nutrition and production growth (Zhang et al., 2004; Lyons, 1973). Especially, the seedling stage is the most susceptible stage for the cultivation of horticultural crops for year-round production in greenhouses, and tomato normally grows at 15 to 33°C, when the external temperature is lower than 10°C, the growth rate slows down (Zhang et al., 2023). Chilling injury is damage caused by exposure to low temperatures stress (0-12°C) during the growing period, which may impair plant growth and productivity (Ronga et al., 2018). Thus, chilling injury is one of the important disasters worth paying attention to in China’s agricultural production research.

Low temperature as one of the main abiotic stresses reduces the cell membrane fluidity and enzyme activities before the temperature reaches a freezing point, inhibits plant physiological metabolic activities, affects seed germination and seedling growth (Feng et al., 2023), and leads to an imbalance between the light energy absorbed by the photosystem and the energy consumed by metabolic reactions.Under natural conditions, plants are exposed to stress sooner or later, and the growth and development of plants depend on and are regulated by mechanisms that absorb light and convert this into usable chemical energy photosynthesis (Valcke, 2021). Photosynthesis is the basic life activity of plants and the fundamental source for plants to synthesize organic matter and obtain energy, and photosynthetic organs are cold-sensitive parts of plants, so low temperatures can directly affect the performance and activity of photosynthesis (Zhang et al., 2022; Liu et al., 2012), such as stomatal conductance, mesophyll conductance, biochemical capacity combined with Rubisco, the Calvin-Benson cycle, thylakoid membrane electron transport, nonphotochemical quenching, and carbon metabolism or fixation pathways (Hou et al., 2016; Hussain et al., 2021).

In recent years, different researchers have applied machine learning and deep learning methods to agricultural data classification, providing a theoretical basis for further improving the industrial automation level of facility agriculture. Hang et al. (2020) identified five common symptoms of tomato diseases by training the Skip gram algorithm, which can be classified using the Long short term memory (LSTM) algorithm. This study showed that the average accuracy of the tomato disease and pest corpus recognition model based on the LSTM algorithm and classifier exceeded 60%. Alkanan and Gulzar (2024) explored the integration of artificial intelligence in maize disease discrimination and classification. This study used a comprehensive dataset and an enhanced iterative network of MobileNetV2 to predict four categories of diseases, with accuracy values ranging from 0.949 to 0.975 and recall values ranging from 0.957 to 0.963. Amri et al. (2024) utilized the advantages of three mature architectures: MobileNet, Inception, and VGG to propose a new deep learning model, MIV PlantNet, specifically designed for classifying different plant communities in Saudi Arabia. The results showed that the accuracy of the MIV PlantNet deep learning model reached 99%, while demonstrating extraordinary accuracy of 96% and excellent F1 performance of 98%, highlighting its robustness and reliability. Gulzar (2024) added five additional layers based on the InceptionV3 architecture, integrating techniques such as transfer learning, adaptive learning rate adjustment (up to 0.001), and model checkpoints to optimize accuracy, and comparative analysis with existing studies reveals competitive accuracy of 98.73% achieved by their proposed model in soybean seed classification. In the research of classification methods for images and data, people are gradually realizing the importance of early recognition of plant classification. Cen et al. (2016) used the hyperspectral imaging system to obtain information about cucumbers, and three classification algorithms of the Naive Bayes(NB), Support Vector Machine(SVM), and K-nearest neighbor (K-NN) method were used to monitor and classify cucumbers into two categories (normal and chilling injury) and three categories (normal, mild, and severe chilling injury) based on the spectrum and image of the selected band ratio. Jung et al. (2022) successfully diagnosed early infection of gray mold on strawberry leaves using hyperspectral images combined with a three-dimensional convolutional neural network (CNN) classification model. They proposed the introduction of a new method that can extract regions of interest (ROIs) from these images to classify different infection states, with a classification accuracy of 84%. Kuo et al. (2023) proposed an improved CNN model, called the 1D-CNN model, which features embedded residual global context (ResGC) blocks and is specifically designed to process Visible spectrum (Vis)/Near Infrared(NIR) spectral data of tomato leaves in order to detect early signs of drought stress. Ünal et al. (2024) detected Vis and NIR images of naturally damaged Super Chief red apples using deep learning models Alexnet, Inception-V3, and Visual Geometry Group(VGG)16 network structures for training and evaluation, and the results showed that when using the NIR dataset for model training and testing, the accuracy of Inception V3 and VGG16 was as high as 100% and 100%, respectively. Therefore, it is recommended to use near-infrared datasets for accurate and reliable apple classification in industrial environments. Meanwhile, the application of chlorophyll fluorescence technology to study plant feature classification is gradually increasing (Cen et al., 2017; Hai-yan et al., 2018). Cen et al. (2017) used chlorophyll fluorescence imaging combined with feature selection to characterize and detect Huanglongbing (HLB) disease. This study combines support vector machines (SVM) and partial least squares discriminant analysis (PLS-DA) classifiers to achieve unique fluorescence features of HLB through three feature selection methods, and to classify healthy, HLB infected, and nutrient deficient leaves for three-level classification. The results showed that this novel data-driven method combined average fluorescence parameters and image features gave the best classification performance, with an accuracy of 97%. Sapoukhina et al. (2022) proposed a new method to generate chlorophyll fluorescence images of diseased plants through automatic lesion annotation. This study mainly segmented disease lesion categories on plant leaf fluorescence images and labeled them accordingly. The results showed that the trained model showed a recall rate of 0.793% and an average accuracy rate of 0.723%. Dong et al. (2020) found that as the stress time increased, the cold damage of tomato seedlings worsened, when the entropy skewness, standard deviation, and color descriptor features b and L/of chlorophyll fluorescence parameter Y (II) were used as input variables for the back propagation neural network (BPNN)l, the prediction accuracy of categories with different degrees of chilling injury under different low temperature stress times was the highest, with an accuracy of 90%. Lu and Lu (2021) obtained 675 nm and 750 nm chlorophyll fluorescence imaging of pickled cucumbers, and the support vector machine(SVM) was used to extract features to categorize the cucumbers into two categories (normal and chilling-damaged) and three categories (normal, mildly and moderately chilling injury), with an overall accuracy of 96.9% and 91.2%, respectively.

**Table 1** summarizes the classification methods of agricultural data from prior literature. Commonly image data types include VIS, hyperspectral, NIR, and chlorophyll fluorescence. VIS-based plant disease and species identification is common, whereas early cold damage classification is less so. Hyperspectral data can detect subtle spectral changes for early warnings, but may be less sensitive than chlorophyll fluorescence during initial cold stress. NIR responds to temperature variations, but subtle temperature changes in crops during early cold damage limit its early detection ability. Chlorophyll fluorescence imaging excels in sensitivity and specificity, accurately reflecting photosynthetic system status, especially PSII reaction center changes, making it a precise indicator of environmental stress including chilling injury.

**Table 1:** Classification methods for agricultural data.

Types of data sources	Research Direction	Classification objective	Research method	Identify initial damage	Reference
Visible spectrum (VIS) image	Disease classification	Five Diseases of Tomatoes	LSTM	/	Hang et al. (2020)
		Four types of corn diseases	MobileNetV2 optimization	/	Alkanan and Gulzar (2024)
	Population classification	Different Plants in Saudi Arabia	MobileNet、Inception and VGG optimization	/	Amri et al. (2024)
	Intraspecific classification	Different seeds of soybeans	InceptionV3 optimization	/	Gulzar (2024)
Hyperspectral image	Environmental coercion classification	Cucumber cold damage level	NB、SVM、K-NN	Yes	Cen et al. (2016)
	Disease classification	Strawberry gray mold	CNN	/	Jung et al. (2022)
Near Infrared (NIR) spectral data	Environmental coercion classification	Tomato drought stress damage	CNN optimization	Yes	Kuo et al. (2023)
	Natural damage classification	Natural damage to apples	Inception V3 and VGG15	/	Ünal et al. (2024)
Chlorophyll fluorescence imaging data	Disease classification	Healthy, HLB infected, and nutrient deficient leaves	SVM and PLS-DA optimization	/	Cen et al. (2017)
		Arabidopsis diseases	U-Net	Yes	Sapoukhina et al. (2022)
	Environmental coercion classification	Tomato chilling injury level	BPNN	Yes	Dong et al. (2020)
		Chilling injury level of pickled cucumber	SVM	Yes	Lu and Lu (2021)

Chlorophyll fluorescence technology is a powerful tool for measuring the photosynthesis of plants and provides the ability to detect damage from a range of biotic and abiotic stressors before visible symptoms appear (Legendre et al., 2021), including chilling injury. Due to the uneven distribution of internal factors in plants, the damage caused by the same stress may also be uneven in space and may have uneven effects on photosynthetic capacity and stomatal aperture, so that different fluorescence activities will be displayed on the same leaf at the same time. Chlorophyll fluorescence imaging (CFI) technology is a visualization technology of chlorophyll fluorescence, which allows studying the spatiotemporal heterogeneity of chlorophyll fluorescence parameters over the entire leaf area (Dong et al., 2019). This technology has become an important method for diagnosing plant photosynthesis and plant stress response mechanism (Herritt et al., 2021; Ogawa and Sonoike, 2021; Kasampalis et al., 2021). Pereira et al. (2011) investigated the color descriptors (Red, Green, Blue, Hue, Saturation, Value, and Lightness) obtained by laser induced fluorescence imaging technology, demonstrating their potential use in monitoring sweet orange greening diseases. By analyzing the average value of color descriptors, fluorescence imaging technology can detect signs of plant disease invasion in the early stages. A new method for detecting Y (II) was developed by Wang et al. (2018) based on chlorophyll fluorescence imaging, which reflects the photosynthetic capacity of plants, R=0.85989, u=0.048803 when using multiple linear regression and R=0.84285, u=0.054739 when using partial least square regression. Dong et al. (2021) used an improved K-means++ clustering algorithm to segment fluorescence images of tomato leaf cold damage. The experimental results showed that the average matching rates of the improved K-means++ algorithm were 0.96%, 13.52%, and 37.08% higher than those of K-means, Fuzzy C-means(FCM), and Hue Saturation Value(HSV) methods, respectively. The average error rates of the improved K-means++ method were 0.16%, 5.56%, and 13.69% lower than those of K-means, FCM, and HSV methods, respectively. These studies indicate that chlorophyll fluorescence technology can be well used to study plant damage. The above classification methods are mostly based on the classification and recognition of agricultural plant species and diseases. while there are relatively few classifications for plant chilling injury under low temperatures, especially in the study of leaf stress damage classification using chlorophyll fluorescence technology on living plants. Previous studies have shown that, with the increase of stress time, the cold damage to tomato seedlings intensifies (Dong et al., 2020). However, in fact, under the same environmental stress, different plants within the same species may have different stress resistance and exhibit varying degrees of damage. How to classify and discuss the degree of damage to tomato seedlings based on their actual damage situation in a targeted manner has become an urgent problem that needs to be solved.

This study focused on tomato seedlings and used dung beetle optimization (DBO) bidirectional(Bi) LSTM to classify and predict the low-temperature cold damage suffered by tomatoes based on chlorophyll fluorescence imaging data. This study had three specific objectives: (1) to classify tomato chilling injury in chlorophyll fluorescence images based on the proportion of the actual cold damage area of each tomato plant in the entire leaf; (2) to establish the BiLSTM model for tomato seedling cold damage based on the image features filtered from each category, and to predict the cold damage category labels of seedlings automatically; (3) to optimize the parameters of the BiLSTM model based on the DBO algorithm and to propose DBO-BiLSTM, which effectively predicted cold damage category labels and improved the accuracy of recognition. This study was expected to detect early chilling injury through chlorophyll fluorescence imaging, achieve automatic classification and labeling of cold damage data, and lay a research foundation for in-depth research on the cold damage resistance of plants themselves and exploring the application of deep learning classification methods in precision agriculture.

## 2 Materials and methods

### 2.1 Sample preparation

The experiment was conducted at the Facility Agricultural Biological Information Testing Laboratory of Shenyang Agricultural University and the Bioinformatics Laboratory of Suqian University between the years 2017 and 2022. The experimental tomato variety used in this study was “L-404”, a cultivar frequently cultivated in Northeast China. Tomato plants were cultivated in peat-based compost within pots, housed in a greenhouse environment. When tomato seedlings reached the 5-leaf, 1-heart stage, 60 vigorous plants were placed in an artificial climate chamber for optimal growth conditions (25°C/12h day, 15°C/12h night, 600μmol m-2 s-1 light, 70% humidity) (Zhang et al., 2014; Dong et al., 2020; Li et al., 2023). Under these conditions, plants grow vigorously. After a week, plants were subjected to low-temperature stress (5°C/12h night for 3 days). Fluorescence imaging data was collected daily at 8 am, preceded by 30-minute dark adaptation before data collection. Chlorophyll fluorescence images were acquired using the MAXI IMAGING-PAM System(Heinz Walz GmbH, Effeltrich, Germany) and processed with the Matlab R2021b and PyCharm Community Edition 2022.

### 2.2 Images features acquisition

In this experiment, all chlorophyll fluorescence images of tomato leaves were collected using the MAXI version of the IMAGING-PAM imaging system (Heinz Walz GmbH, Effect, Germany). The device uses 44 parallel optical corrected ultra strong emitting blue (450 nm) diodes as the excitation light source, and measures once every 24 hours for 3 consecutive days.All of which were collected from live leaves. When collecting data, the leaves did not leave the plant and continued to exhibit photosynthetic activity. The artificial climate chamber and IMAGING-PAM were placed in the same experimental greenhouse, and during the experiment collection, the greenhouse was in a completely black state to eliminate the potential impact of light. IMAGING PAM obtains chlorophyll fluorescence of plant leaves, generates fluorescence grayscale image, and displays them as color image through pseudo coloring. The 36 features of chlorophyll fluorescence images were processed and obtained by the program, including 18 color image features and 18 gray image features.

First, 18 features were extracted from chlorophyll fluorescence color images of healthy and chilling injury leaves, of which nine color descriptor features (R, G, B, H, S, V, L, a, b) were from RGB, HSV, and L*a*b* color spaces, while the other nine features were their ratios (parameter feature columns in **Table 2**). Subsequently, eighteen features was extracted from the leaf gray image, from chlorophyll fluorescence gray images of healthy and chilling injury leaves, encompassing four statistical features (mean, standard deviation, skewness, and smoothness, as shown in **Table 3**) derived from the histogram, six texture features (low gradient advantage、high gradient advantage, non-uniformity of gray distribution, non-uniformity of gradient distribution, average gradient, gradient standard deviation, as shown in **Table 4**) extracted from the Gray-level Gradient Co-occurrence Matrix (GGCM), and an additional four texture features (energy, entropy, inertia, and correlation, as shown in **Table 5**) along with their respective standard deviations, sourced from the Gray-Level Co-occurrence Matrix (GLCM) (Qiao et al., 2019; Dong et al., 2020).

**Table 2:** Table of spearman correlation.

Characteristic parameters	Spearman Correlation	Characteristic parameters	Spearman Correlation	Characteristic parameters	Spearman Correlation
R	0.338	H	0.417	L	0.557
G	0.540	S	0	a	-0.465
B	0.076	V	0.381	b	0.555
G/R	0.496	S/H	-0.417	L/a	0.464
G/B	-0.076	V/S	0.380	L/b	0.496
B/R	0.076	V/H	-0.352	b/a	-0.089
Mean	0.893	Homogeneity of gray distribution	-0.835	Inertia	0.204
Standard deviation	0.885	Homogeneity of gradient distribution	-0.826	Mean of correlation	0
Skewness	0.802	Average gradient	0.803	Standard deviation of energy	0.147
Histogram Smoothness	0.884	Gradient standard deviation	0.750	Standard deviation of entropy	0.221
Small gradient dominance	-0.824	Mean of energy	-0.312	Standard deviation of inertia	0.190
High gradient advantage	0.803	Mean of entropy	0.309	Standard deviation of correlation	0

**Table 3:** Statistical features of gray image.

Characteristic Parameters	Definition	
$E=\sum_{i=1}^{N_{g}}iH\left(i\right)$	Mean, reflect the overall brightness of the image.	Where *i* is gray level; *N_g_* is the number of gray levels, *H(i)* is the discrete function of image gray level.
$\sigma =\sqrt{\frac{1}{N_{g}}\sum_{i=1}^{N_{g}}{\left(i-E\right)}^{2}}$	Standard deviation, reflects the extent to which the pixel value is offset from the mean.	
$S=\sqrt[{3}]{\frac{1}{N_{g}}\sum_{i=1}^{N_{g}}(i-E)3}$	Skewness, reflecting the symmetry of the image color distribution.	
$R=1-\frac{1}{1+\sigma^{2}}$	Smoothness, measure of the relative smoothness of grayscale within an image.	

**Table 4:** Texture features from the GGCM of gray image.

Characteristic Parameters	Definition	
$T_{1}=\frac{\sum_{i=1}^{N_{g}}\sum_{j=1}^{T}H(i,j)}{H}$	Low gradient advantage, reflects the degree of dominance of pixels with smaller gradient values in the image.	Where *H(i,j)* in the gray gradient co-occurrence matrix indicates the probability of pixel pairs with gray level *i* and gradient level *j*. *H* represents the total sum of these elements. *T*, a gradient threshold, differentiates “low” from “high” gradients, classifying gradients exceeding *T* as “High.”.
$T_{2}=\frac{\sum_{i=1}^{N_{g}}\sum_{j=\text{T}+1}^{N_{s}}j^{2}H(i,j)}{H}$	High gradient advantage, reflects the proportion of areas with drastic grayscale changes in the image.	
$T_{3}=\frac{\sum_{i=1}^{N_{g}}{\left[\sum_{j=1}^{N_{s}}H(i,j)\right]}^{2}}{H}$	Non-uniformity of gray distribution.	
$T_{4}=\frac{\sum_{j=1}^{N_{s}}[{\sum_{i=1}^{N_{g}}H(i,j)]}^{2}}{H}$	Non-uniformity of gradient distribution.	
$\mu_{=\sum_{j=1}^{N_{s}}j[\sum_{i=1}^{N_{g}}P(i,j)]}$	Average gradient.	Where *P(i,j)* signifies the normalized pixel probability, reflecting image clarity and texture depth. *N_g_* and *N_s_* represent the number of gray levels and gradient levels, respectively.
∂={∑j=1Ns(j−μ2)2[∑i=0NgP(i,j)]}12	Gradient standard deviation.	

**Table 5:** Texture features from the GLCM of gray image.

Characteristic Parameters	Definition	
$ASM=\sum_{i}\sum_{j}\left[Q{\left(i,j\right)}^{2}\right]$	Energy, it is used to measure the degree of texture thickness of the image.	Where *i*, *j* represent grayscale levels of points, *Q(i,j)* is the normalized grayscale matrix. *μ_x_*, *μ_y_* are average grayscale, gradient, *σ_x_*, *σ_y_* are their standard deviation, respectively.
$ENT=\sum_{i}\sum_{j}Q\left(i,j\right)\log Q\left(i,j\right)$	Entropy, it describes the degree of complexity of an image.	
$INE=\sum_{i}\sum_{j}{\left(i-j\right)}^{2}Q\left(i,j\right)$	Inertia,it reflects the periodicity of texture changes.	
$COR=\frac{\sum_{i}\sum_{j}\left(i\times j\right)Q\left(i,j\right)-\mu_{x}\mu_{y}}{\sigma_{x}\sigma_{y}}$	Correlation, it indicates the degree of similarity of the gray level co-occurrence matrix in the row or column direction.	

### 2.3 Classification method for chilling injury

At present, there is no clear classification method for the degree of chilling injury under low- temperature stress. The degree of damage in crop diseases is generally expressed by the percentage of the diseased area in the total leaf area or the average diameter of the diseased area (Qiao et al., 2019; Sun et al., 2018). Compared with the disease damage, the chilling injury may be distributed at any position of the leaf, and sometimes it is difficult to calculate the average diameter when the chilling injury area is scattered. Therefore, this study divided the chilling injury classification according to the above method of the percentage of damage area to total leaf area, and all leaves were divided into sound leaves, slight chilling injury leaves, moderately chilling injury leaves, and severe chilling injury leaves. Since the area of chilling injury was proportional to the number of pixels in the chilling injury area, and the area of chilling injury leaf was proportional to the number of pixels in the chilling injury leaf area, the classification of the degree of chilling injury could be expressed by the ratio *L_k_* of the number of pixels in the chilling injured area to the total number of pixels in the leaf area, as shown in Equation 1 (Wang et al., 2021).


(1) $$L_{k}=A_{1}/A=N_{1}/N$$

Where in, *A1* was the area of the chilling injury area, and *A* was the total area of the chilling injury leaf; *N1* was the number of pixels in the chilling injury area, and *N* was the total number of pixels in the chilling injury leaf. Chlorophyll fluorescence image chilling injury area can be calculated via threshold segmentation of chilling injury area ratios (Souza and Yang, 2021).

### 2.4 Spearman rank correlation coefficient

Spearman rank correlation coefficient is an indicator that describes whether there is equal or opposite convergence between two groups of variables (Spearman, 1904). The Spearman correlation coefficient only needs to determine the level at each point (period) to obtain the correlation, so it has good properties. Under the condition that there are no duplicate observations in both groups of data, the equation Equation 2 Spearman rank coefficient is used to determine the statistical correlation between different characteristics and chilling injury (Amarkhil et al., 2021).


(2) $$R_{s}=1-\frac{6\sum d_{i}^{2}}{n^{3}-n}$$

Where, $R_{s}$ = Spearman rank correlation coefficient ( $R_{s}$ value ranging from 1 showing the robust direct relationship, and -1 showing strong inverse correlation), $d_{i}$ = difference in causes ranking of the identified conditions, *n*= number of variables (Amarkhil et al., 2021). Currently, despite a lack of a unified classification criterion, the degree of correlation strength between variables is frequently employed as a basis for categorization (Song and Park, 2020; Jia et al., 2020; Amarkhil et al., 2021). In this experiment, the strength of the correlation was described using the following guide for the absolute value of $R_{s}$ : 0-0.29,”weak”; 0.30-0.59, “moderate”; 0.50-0.79, “strong”; and 0.80 to 1.0, “very strong” (Dong et al., 2020; Yu and Hutson, 2024).

### 2.5 Bidirectional long short term memory network

Due to the time series relationship between different classifications and features of chlorophyll fluorescence images of tomato seedlings, this study chose Long Short Term Memory (LSTM) network.

#### 2.5.1 LSTM network

**Figure 1:**
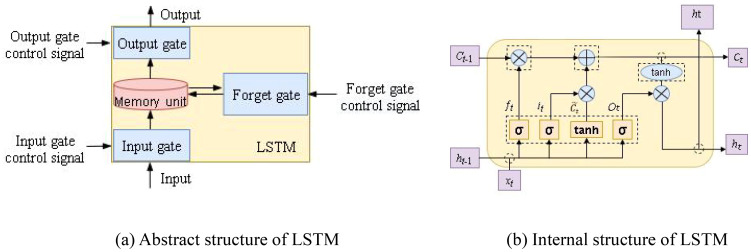
1 LSTM structure. **(A)** Abstract structure of LSTM. **(B)** Internal structure of LSTM.

Long short term memory (LSTM) networks store “memory” in a special way (Devi and Thongam, 2023).They were first proposed by Hochreiter and Schmidhuber in 1997, which initially introduced the clever concept of self circulation, providing an efficient path to achieve long-term continuous transmission of gradients (Liu et al., 2018). **Figure 1A** shows an abstract diagram of the LSTM neural network structure, explaining its memory method. The gate structure of LSTM neural network can control the inflow and outflow of information, which is divided into three stages: forget gate, input gate, and output gate. The LSTM forget gate controls which information in a memory cell needs to be retained and which needs to be forgotten, by means of a sigmoid function and a tanh function. The input gate controls which new information can flow into the memory cell by means of a sigmoid function and a tanh function. The output gate controls which information can affect the current output through a sigmoid function.

**Figure 1B** shows the internal structure of LSTM. The input is to store external content into a memory unit, and the main function of the input gate control signal is to control the input gate, denoted as $i_{t}$ . The main function of the forget gate control signal is to control whether the memory unit is cleared, denoted as $f_{t}$ . The output mainly refers to the content of the memory unit being output to other hidden layers, and the function of the output gate control signal is mainly to control whether the memory unit is output, denoted as $O_{t}$ . LSTM can store important content in memory unit $C_{t}$ , achieve functions such as outputting or clearing when needed, and construct “long-term dependencies” through memory units, and “short-term dependencies” through hidden layer states $h_{t}$ . Among them, $x_{t}$ represents the input of the neuron, σ represents the sigmoid excitation function, *tanh* represents the *tanh* excitation function, $\tilde{C_{t}}$ represents the new candidate values created by the excitation function *tanh* that can be added to the cell unit, ⊗ represents the multiplication operation, ⊕ represents the addition operation, and → represents the transfer of vectors.

#### 2.5.2 Bi LSTM network

**Figure 2:**
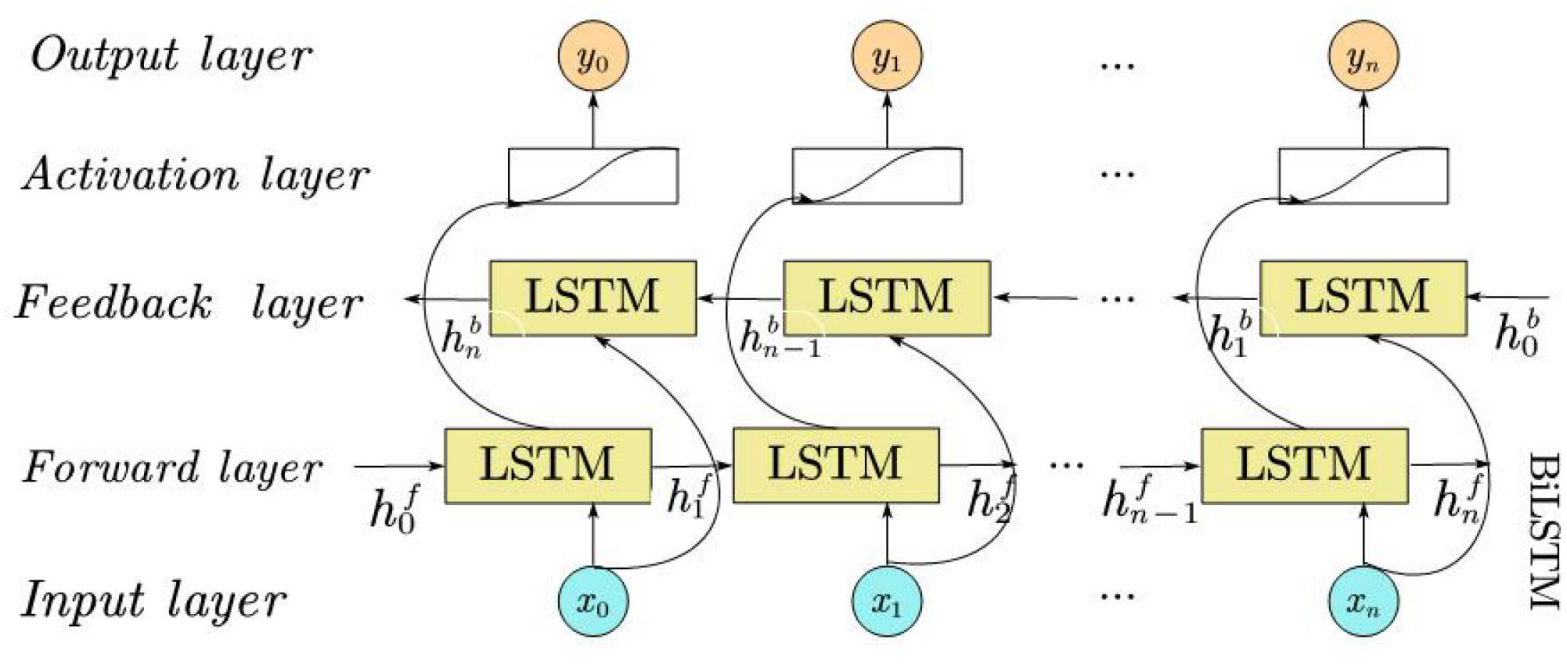
BiLSTM network structure.

The traditional LSTM network calculates sequence data in chronological order, step by step from front to back. However, in some cases, the backward dependency relationship in the sequence is also very important, that is, the output of one time step may also be affected by subsequent time steps. To solve this problem, the bidirectional LSTM (BiLSTM) network added an inverse layer to achieve bidirectional scanning, which can capture more contextual information in a single model and achieve better prediction, as shown in **Figure 2**.

BiLSTM consists of a forward LSTM layer and a reverse LSTM layer (Michael et al., 2024). Combinations of hidden states in both directions, which are fed into other layers, can be used to extract features in the time dimension. The BiLSTM summarizes information by concatenating the forward (left-to-right, Equation 3) and backward (right-to-left, Equation 4) hidden states, yielding its output as per Equation 5 (Hameed and Garcia-Zapirain, 2020).


(3) $$\vec{h_{t}^{f}}=LSTM\left(h_{t-1},x_{t-1}\right)$$

(4) $$\overset{\leftarrow }{h_{t}^{b}}=LSTM\left(h_{t+1},x_{t-1}\right)$$

(5) $$h_{t}=\vec{\alpha h_{t}^{f}}+\overset{\leftarrow }{\beta h_{t}^{b}}$$

Where, $x_{t}$ , $\vec{h_{t}^{f}}$ , $\overset{\leftarrow }{h_{t}^{b}}$ are the input data of the moments, the output of the forward LSTM hidden layer and the output of the reverse LSTM hidden layer, respectively. ${\text{h}}_{\text{t}}$ denotes the combination of $\vec{h_{t}^{f}}$ and $\overset{\leftarrow }{h_{t}^{b}}$ at moment *t*, α,β are constant coefficients and denote the weights of and $\vec{h_{t}^{f}}$ , $\overset{\leftarrow }{h_{t}^{b}}$ respectively.

The working steps of the BiLSTM network for predicting chilling injury to tomato leaves were summarized as: data preparation, construction of the BiLSTM model, model initialization, model training, model prediction, and evaluation. The input data of the model was the chlorophyll fluorescence image of tomatoes under low temperature stress.

### 2.6 Dung beetle optimization - BiLSTM

#### 2.6.1 DBO algorithm

The Dung beetle optimization (DBO)-BiLSTM algorithm incorporates the population intelligence optimization technique known as DBO (Dung beetle optimizer), which draws inspiration from the behaviors of dung beetles, specifically their rolling and reproductive habits. This approach was initially introduced in 2022 (Xue and Shen, 2023).

During the rolling process, dung beetles rely on celestial cues to navigate and ensure that the dung ball rolls in a straight path. To simulate this behavior, it is assumed that the beetles move in a predetermined direction throughout the entire search space. As dung beetles use the sun for navigation, it is also assumed that the intensity of the light source impacts their path. The update of the beetle’s position during rolling can be represented as Equations 6, 7 (Byrne et al., 2003):


(6) $$x_{i}\left(t+1\right)=x_{i}\left(t\right)+\alpha *k*x_{i}\left(t-1\right)+b*\Delta x$$

(7) $$\Delta x=\left|x_{i}\left(t\right)-X^{\omega }\right|$$

Where, t denotes the current iteration number, ${\text{x}}_{\text{i}}\left(\text{t}\right)$ denotes the position information of the i moult at the t iteration, k∈(0,0.2] denotes the deflection coefficient, k is a fixed value,b denotes a fixed value belonging to (0,1), α is a natural coefficient assigned to -1 or 1, ${\text{X}}^{\omega }$ denotes the global worst position, Δx is used to simulate the change of light intensity.

1. In nature, dung beetles choose a safe environment as a place to lay eggs. The strategy of simulating dung beetles to select the boundary of the spawning area are defined as Equations 8, 9:


(8) $$Lb^{*}=max\left(X^{*}\times \left(1-R\right),Lb\right)$$

(9) $$Ub^{*}=min\left(X^{*}\times \left(1-R\right),Ub\right)$$

2. Among them, $X^{*}$ is the current local optimal position, $Lb^{*}$ and $Ub^{*}$ are the lower and upper bounds of the spawning area, $R=\frac{1-t}{T_{max}}$ and $T_{max}$ represent the maximum number of iterations, Lb and Ub represent the upper and lower bounds of the optimization problem, respectively.

3. In the DBO algorithm, the female dung beetle produces only one egg in each iteration. According to formula (8) and (9), the spawning range is influenced by the value of *R*, resulting in the egg’s location changing. This is defined as Equation 10:


(10) $$B_{i}\left(t+1\right)=X^{*}+b_{1}\times \left(B_{i}\left(t\right)-Lb^{*}\right)+b_{2}\times \left(B_{i}\left(t\right)-Ub^{*}\right)$$

Where, ${\text{B}}_{\text{i}}\left(\text{t}\right)$ is the position information of the i sphere at the t iteration, ${\text{b}}_{1}$ and ${\text{b}}_{2}$ are denoted as two independent random vectors of size 1 × D, with D denoting the number of dimensions, and the position of the sphere is strictly limited to a certain range (Mirjalili and Lewis, 2016).

4. The larvae that grow out of the egg globule, which we call oyster roach, need to establish the optimal feeding zone to guide the oyster roach to feed in the DBO algorithm, and the optimal feeding zone are defined as Equations 11, 12:


(11) $$Lb^{b}=max\left(X^{b}\times \left(1+R\right),Lb\right)$$

(12) $$Ub^{b}=min\left(X^{b}\times \left(1+R\right),Ub\right)$$

Where, $X^{b}$ is the global optimal feeding position, $Lb^{b}$ and $Ub^{b}$ are the lower and upper bounds of the optimal feeding zone, respectively.

5. Thus the position of the oyster roach is updated, defined as Equation 13:


(13) $$x_{i}\left(t+1\right)=x_{i}\left(t\right)+C_{1}\times \left(x_{i}\left(t\right)-Lb^{b}\right)+C_{2}\times \left(x_{i}\left(t\right)-Ub^{b}\right)$$

Where ${\text{x}}_{\text{i}}\left(\text{t}\right)$ denotes the position of the i oyster roach at the t iteration, ${\text{C}}_{1}$ denotes a random number obeying a normal distribution, ${\text{C}}_{2}$ denotes a random vector belonging to (0,1).

6. Dung Beetle iterates the position updates in a continuous optimization process and finally outputs the optimal position as $X^{b}$ (Xue and Shen, 2023).

#### 2.6.2 DBO-BiLSTM model

Traditional BiLSTM networks require adjusting a large number of parameters during the training process, such as learning rate, number of hidden layer units, regularization parameters, etc. The adjustment of these parameters often relies on manual experience and trial and error methods, which are inefficient and have unstable effects. In the experiment, after the data preparation of the DBO-BiLSTM model in the working steps of the BiLSTM network, the hyperparameters of the BiLSTM model were optimized using the Dung Beetle Algorithm (DBO) (Xue and Shen, 2023) before constructing the model.The DBO algorithm can simulate the behaviors of dung beetles such as rolling, dancing, foraging, stealing, and breeding. Through iterative optimization, it can find the optimal solution of network parameters, avoid getting stuck in local optima, and improve the training efficiency and performance of BiLSTM networks. The main optimization parameters in this study are: learning rate, number of hidden layer nodes, and regularization coefficient. The specific optimization steps were as follows:

1. Initialized the dung beetle population: Randomly initialized a group of dung beetles in the search space, with each beetle representing a set of hyperparameters.
2. Evaluated hyperparameter combinations: Trained BiLSTM models with each set of hyperparameters and evaluated the performance of the models (such as accuracy, loss, etc.) on the test sample set. This evaluation result served as the fitness value for that group of hyperparameters.
3. Updated the positions of the beetles: Based on their fitness values and search strategies (such as random walking, following the optimal solution, etc.), updated the positions of the beetles in the search space, that is, updated the values of the hyperparameters.
4. Iteratively optimized: Repeated the steps of evaluating hyperparameter combinations and updating dung beetle positions until the termination conditions were met (such as reaching the maximum number of iterations, convergence of fitness values, etc.).
5. Selected the optimal hyperparameter combination: After the optimization process was completed, selected the hyperparameter combination represented by the beetle with the highest fitness value as the optimal solution.
6. Trained the BiLSTM model using optimal hyperparameters: Constructed and trained the BiLSTM model using the selected optimal hyperparameter combinations.

#### 2.6.3 Confusion matrix evaluation

The confusion matrix is used to evaluate the DBO-BiLSTM model. The evaluation criteria are shown in **Table 6**. The model finally needs to judge whether the result of the sample is 0 or 1, or positive or negative.

**Table 6:** The confusion matrix values.

The confusion matrix		True values	
		Positive	Negative
Predicted values	Positive	TP	FP (Type II)
	Negative	FN (Type I)	TN

**Table 6** presents the confusion matrix classification. True Positive (TP) denotes correctly predicted positive samples, True negative (TN) represents correctly predicted negative samples, False positive (FP) indicates incorrectly predicted negative samples as positive, and False negative (FN) signifies incorrectly predicted positive samples as negative (Valero-Carreras et al., 2023).

When the values of TP and TN are large and the values of FP and FN are small, it indicates a better predictive classification model. However, it is difficult to measure the superiority of the model when dealing with a large amount of data. Therefore, the confusion matrix extends the following four indicators based on the basic statistical results to measure the performance of the classification model. Accuracy is the proportion of all correct predictions in the classification model to the total number of test samples, as shown in Equation 14; Precision is the correct proportion of results that are positive, and the denominator is the sum of all values in this column, as shown in Equation 15; Sensitivity, also known as recall, is the proportion of the classification model with positive true values and correct predictions, with the denominator representing the total number of all true positive samples, as shown in Equation 16; F1-measure is the reconciled mean of precision and recall, which is a comprehensive index considering the balance between the two, distributed between [0,1], the closer to 1 the better, as shown in Equation 17 (Xiong et al., 2022).


(14) $$Accuracy=\frac{TP+TN}{TP+TN+FP+FN}$$

(15) $$Precision=\frac{TP}{TP+FP}$$

(16) $$Recall=\frac{TP}{TP+FN}$$

(17) $$F1-measure=\frac{2}{\frac{1}{Precision}+\frac{1}{Recall}}=\frac{2*Precision*Recall}{Precision+Recall}$$

## 3 Results

### 3.1 Classification based on chilling injury areas

Chlorophyll fluorescence imaging technology can reflect the internal photosynthetic capacity of plants through photosynthetic physiological parameters and their activity distribution, and can detect early cold damage before it is visible to the naked eye.

**Figure 3:**
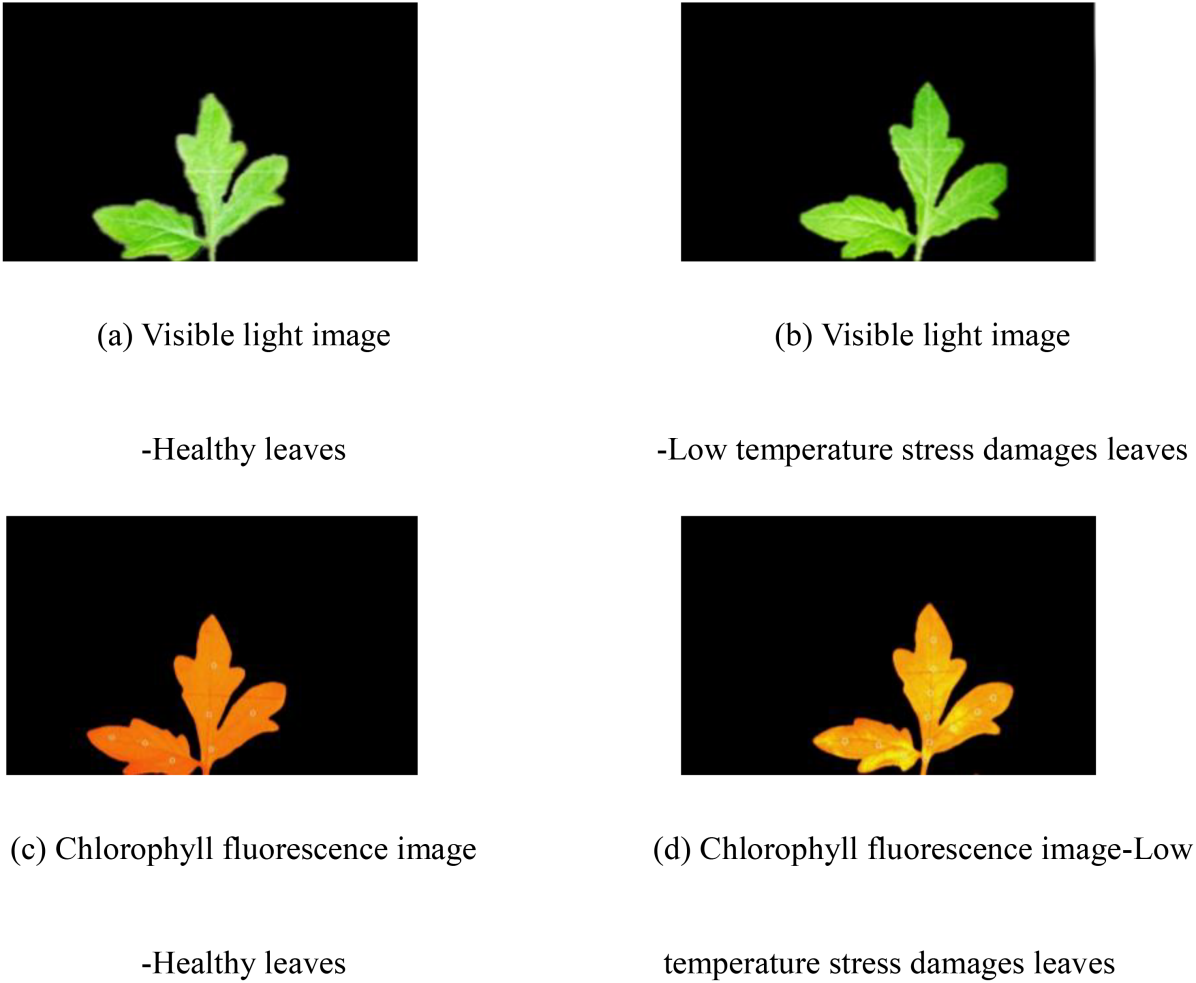
Image of tomato leaves. **(A)** Visible light image of healthy leaves. **(B)** Visible light image of leaves damaged by low temperature stress. **(C)** Chlorophyll fluorescence image of healthy leaves. **(D)** Chlorophyll fluorescence image of leaves damaged by low temperature stress.

**Figure 3** displays the visible light and chlorophyll fluorescence images of tomato seedling leaves. **Figures 3A, C** depict healthy leaves, whereas **Figures 3B, D** represent the leaves exposure to low temperature stress. As evident from the figure, chlorophyll fluorescence imaging is capable of detecting early damage prior to it becoming visually apparent, underscoring its potential for early stress detection. According to Equation 1, the proportion of chilling injury areas was used to classify chlorophyll fluorescence images. The classification method of chilling injury to tomato seedling leaves was shown in **Table 7**.

**Table 7:** Classification of chilling injury tomato.

Classification	Chilling injury level	Level standard	Labels
1	Sound	*L_k_<5%*	A
2	Slight chilling injury	*L_k_<15%*	B
3	Moderate chilling injury	*L_k_<30%*	C
4	Severe chilling injury	*L_k_>30%*	D

The experimental data categorized tomato seedling leaves into four levels based on the percentage of chilling injury: Level 1 (0-5%, healthy), Level 2 (5-15%, slightly damaged), Level 3 (15-30%, moderately damaged), and Level 4 (>30%, severely damaged). During the experiment, Level 1 corresponded to A, Level 2 to B, Level 3 to C, and Level 4 to D.

### 3.2 DBO-BiLSTM identifies chilling injury classification

#### 3.2.1 Input feature selection of DBO-BiLSTM

After classifying the chlorophyll fluorescence images of tomato leaves, 220 images were selected, which included four types of images: A, B, C, and D. 36 features were extracted from each image, of which 18 were from color images and 18 were from grayscale images. Due to the fact that each image has 36 features, which are input into the classification model for detection, the computational workload is high. In order to perform effective calculations, it was necessary to reduce the dimensionality of 36 features. Although the principal component analysis (Bro and Smilde, 2014) well reduced the dimension of features, it couldn’t retain the original information parameters, so Spearman feature selection (Amarkhil et al., 2021) was used to analyze features. The formula (2) in the previous 2.4 was used to calculate the Spearman correlation between the characteristics of the chlorophyll fluorescence grayscale image and the chilling injury classes of tomato leaves, as shown in **Table 2**.

Among them, the correlations between chilling injury levels and the histogram mean value, histogram mean square deviation, histogram third moment, histogram smoothness, Low gradient advantage, large gradient advantage, non-uniformity of gray distribution, gradient distribution non-uniformity, and gradient mean value were between 0.8-1.0, which was the very strong correlation group, and defined as cold sensitive-feature group; The correlations between the degree of chilling injury and the mean square differ- ence of L, B, and gradient were between 0.5 and 0.8, which was the strong group; The correlations bet- ween the chilling injury levels and R, G/R, H, V, S/H, V/S, V/H, a, L/a, L/b, energy average, and entropy average were between 0.3-0.5, which was the moderate group; The correlations between the chilling injury levels and B, G/B, B/R, S, b/a, inertial mean, correlation mean, energy standard deviation, entropy standard deviation, inertial standard deviation, and correlation standard deviation were between 0.0-0.3, which was the weak group. Finally, the very strong group with a high correlation was selected to identify the chilling injury classifications in tomato leaves,that is, select the following features as input features for the DBO-BiLSTM prediction model: histogram mean, histogram mean square error, histogram third moment, histogram smoothness, Low gradient advantage, large gradient advantage, grayscale distribution non- uniformity, gradient distribution non-uniformity, and gradient mean.

#### 3.2.2 DBO-BiLSTM identifies chilling injury classification

The 220 images in the chlorophyll fluorescence image set were divided into two groups in an 8:2 ratio, with 176 images in the training set and 44 images in the prediction set. The training set and test set samples were input into the model for training and prediction, respectively. This experiment adopted the BiLSTM algorithm in deep learning and optimized it with DBO to achieve deep learning for chilling injury recognition and classification of tomato seedlings. In the BiLSTM model, the number of hidden nodes is 200, the initial learning rate is 0.001, and the regularization parameter is 0.001. Optimize the BiLSTM model using dung beetles, optimize three parameters based on initial values, and determine the optimal values of the number of hidden nodes in the model, as well as the learning rate and regularization parameters of the model parameters.

As show in **Table 8** The experimental input data is the characteristic parameters of chlorophyll fluorescence parameter F image, with a total of 220 images, including 4 groups of chilling injury classification (chilling injury level 1, level 2, level 3, and level 4). Among them, there were 176 training samples, and the number of chlorophyll fluorescence images corresponding to 1, 2, 3, and 4 levels of chilling injury leaves was 42, 46, 46, and 42, respectively, and the labels were A, B, C, and D, respectively. There were 44 test samples, and the number of chlorophyll fluorescence images corresponding to 1, 2, 3 and 4 chilling injury leaves was 10, 12, 12 and 10, respectively, and the labels were A, B, C and D, respectively. The training set sample leaves were coded, and 1~42 was the “Sound” group; 43~88 was the “Slight chilling injury” group; 89~134 was the “Moderate chilling injury” group; 135~176 was the “Severe chilling injury” group. The sample leaves of the test set were numbered, and 1~10 was the “Sound” group; 11~22 was the “Slight chilling injury” group; 23~34 was the “Moderate chilling injury” group; 35~44 was the “Severe chilling injury” group.

**Table 8:** Experimental images classification.

Classification	Number of images in the training set	Coded for the images in the training set	Number of images in the test set	Coded for the images in the test set
1	42	1~42	10	1~10
2	46	43~88	12	11~22
3	46	89~134	12	23~34
4	42	135~176	10	35~44
	176		44	

**Figure 4:**
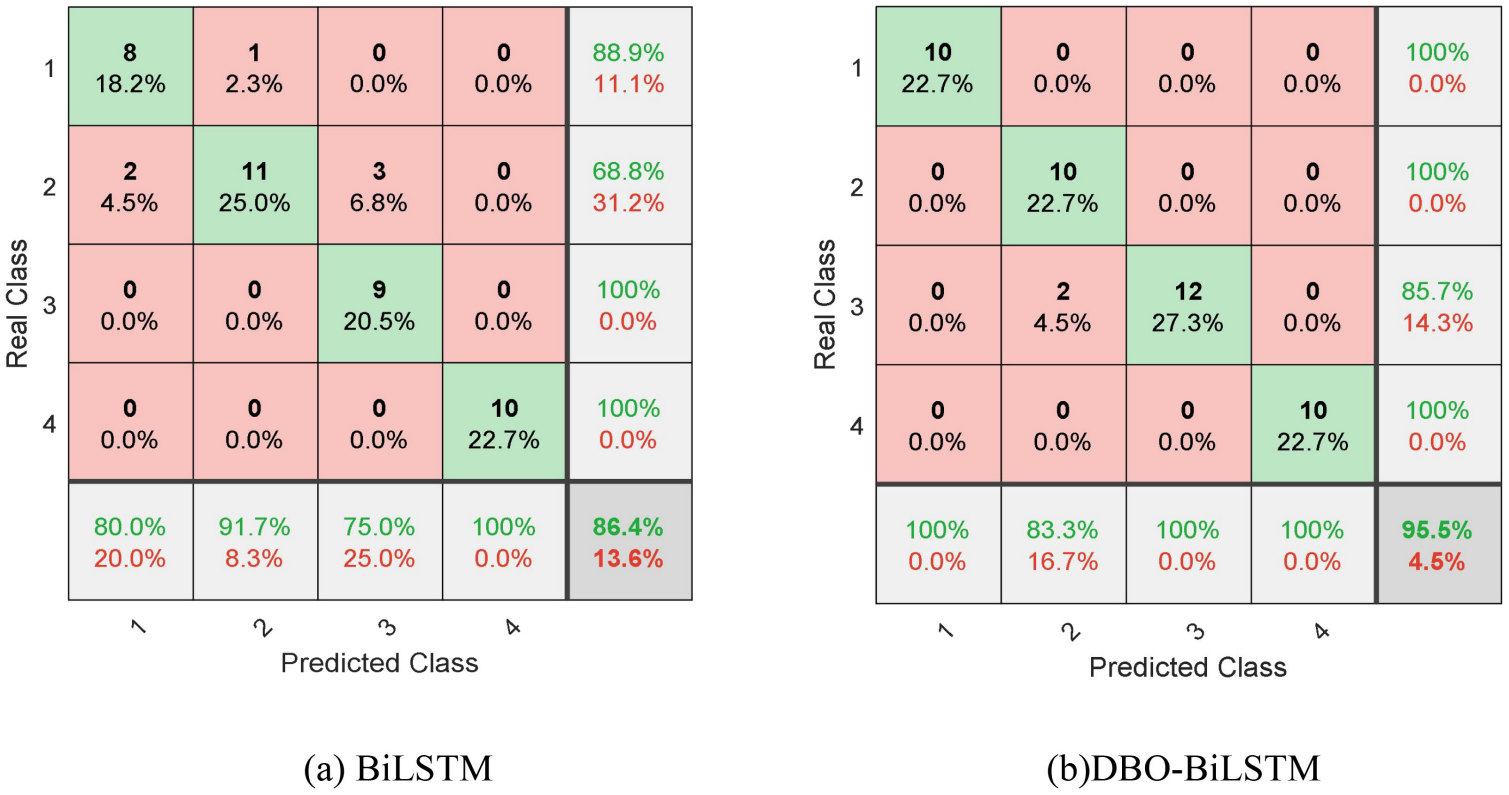
Confusion matrices for classifying and identifying chilling Injury in tomato seedlings. **(A)** Confusion matrix obtained from the BiLSTM model. **(B)** Confusion matrix obtained from the DBO-BiLSTM model.

The group features with a strong relationship with cold damage were selected based on the Spearman rank correlation coefficient, as input vectors, and the recognition performance of BiLSTM model and DBO-BiLSTM mode for low-temperature damage classification was compared. After many experiments, the optimal parameter setting value is preliminarily determined: the population size is 20, the number of iterations is 8, and the maximum training times is 500. This article uses the DBO algorithm to optimize the parameters of the BiLSTM model. The learning rate reduction factor is 0.1, the learning rate range is set between 0.0001 and 0.1, the number of hidden layer nodes is selected between 5 and 100, and the regularization parameter range is 0.0001 to 0.1. Through search, the best hyperparameter combination is automatically found. The model evaluation and cold damage identification results of BiLSTM model and DBO-BiLSTM model are shown in **Figure 4**.

As shown in **Figure 4A**, each column of the BiLSTM confusion matrix represents the prediction category, the total number of each column represents the number of predicted data for this category; each row represents the actual data category, and the total number of data per row represents the actual number of data for that category. The actual corresponding numbers of chilling injury labeled as categories 1, 2, 3 and 4 were 10, 12, 12 and 10, respectively, while the number of correct predictions was 8 in category 1, 11 in category 2, 9 in category 3 and 10 in category 4, respectively, as shown in the green part of **Figure 4A**. Category 1 contained 8 positive and 2 negative samples and had 80% accuracy in recognizing healthy leaves. In the second category, there were 11 positive samples and 1 negative sample, and the recognition accuracy of leaves with mild chilling injury reached 91.7%. The third category contained 12 samples, including 9 positive samples and 3 negative samples, and the recognition accuracy of leaves with moderate chilling injury was 75%. Category 4 contained 10 samples, and all severe chilling injury samples were correctly predicted. That is, in the first category, two leaves were misdiagnosed as cold damage in the second category. In the second category, one leaf was misdiagnosed as cold damage in the first category. In the third category, three leaves were misdiagnosed as chilling injury in the second category.

After improvement, in the DBO-BiLSTM confusion matrix model, the actual corresponding numbers of chilling injury marked as categories 1, 2, 3, and 4 in **Figure 4B** are 10, 12, 12, and 10, respectively. The number of correct predictions is 10 for Category 1, 10 for Category 2, 12 for Category 3 and 10 for Category 4, respectively, as shown in the green section of **Figure 4B**. In the second category, two leaves were misdiagnosed as the third category. The DBO-BiLSTM proposed in this paper improved the recognition accuracy of moderate chilling injury by 25% relative to the BiLSTM, but shows a larger error in the classification prediction of slight chilling injury. The reason for misdiagnosis may be that the characteristics of chilling injuries are close to each other, or the incomplete segmentation of chilling injuries is not accurate enough.

**Figure 5:**
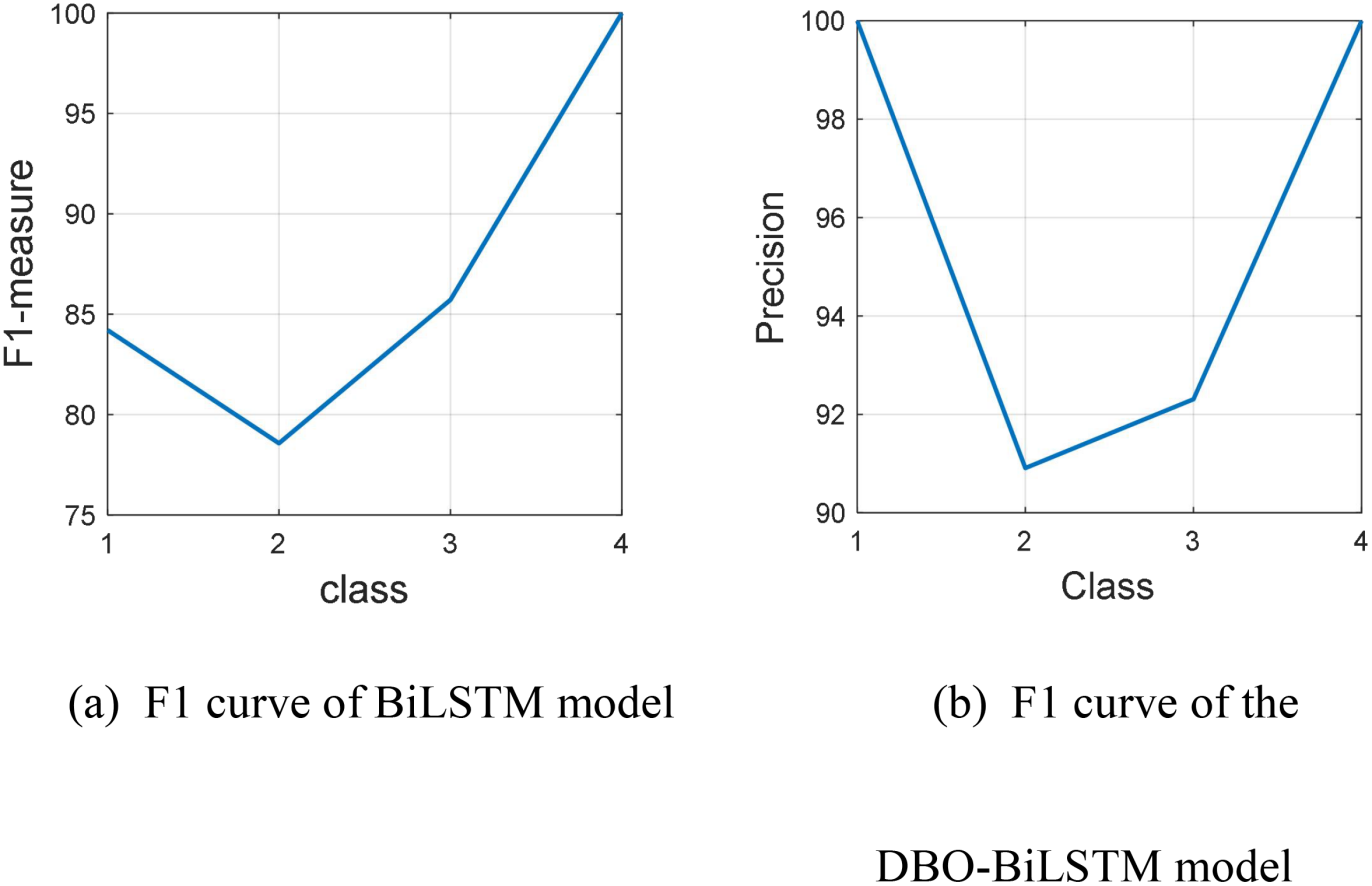
F1 Curve Analysis. **(A)** F1 Curve of the BiLSTM Model. **(B)** F1 Curve of the DBO-BiLSTM Model.

The F1 measure is the harmonic mean of accuracy and recall, commonly used as a comprehensive indicator for model evaluation. The line chart intuitively displays the changes in F1 score as the classification changes. **Figure 5A** shows that the F1 measurement values of the BiLSTM model in four categories are 84, 79, 86, and 100, with values higher than 75 but lower than 100. It is evident that the recognition of tomato leaf cold damage in categories 1 and 4 is relatively good, and even the F1 score reaches 100% in severe cold damage categories. However, the recognition performance of tomato leaf cold damage in categories 2 and 3 is poor. **Figure 5B** shows that the F1 measurement values of the BiLSTM model optimized by DBO are 100, 91, 92, and 100 in all four categories, with all values ranging from 90 to 100. Obviously, after optimizing the BiLSTM model by DBO module, the recognition performance of healthy, mild, and moderate cold damage categories is significantly improved, and the recognition of severe cold damage categories is 100%, just like the BiLSTM model. Compared to the BiLSTM model, the improved DBO-BiLSTM model showed an increase in F1 scores. The DBO-BiLSTM model increased scores by 16, 12, 6, and 0 points in categories 1, 2, 3, and 4, respectively. Therefore, the improvement effect of BiLSTM model in tomato seedling cold damage classification using chlorophyll fluorescence imaging is significant. However, both models showed slightly lower performance in identifying mild and moderate cold damage, but showed higher accuracy in identifying healthy and severe cold damage tomato seedlings, with severe cold damage reaching up to 100 points. This may be due to the ease of distinguishing between healthy and severe cold damage in image data, while identifying mild and moderate cold damage is relatively difficult.

## 4 Discussion

In the experiment, in the F-parameter images captured by the IMAGING-PAM fluorescence imager, ordinary tomato leaves appeared red, the background at non-leaf positions appeared black, and the cold damaged areas appeared yellow-green. The above characteristics enhanced the contrast between the target and background, which was beneficial for effective image segmentation and recognition. After low temperature stress, tomato seedlings showed significant horizontal heterogeneity distribution changes in leaf green fluorescence imaging, which is consistent with the study of plant chlorophyll fluorescence damage heterogeneity by Hai-yan et al. (2018). Models such as CNN (Jung et al., 2022), Alexnet, Inception-V3, and VGG16 (Ünal et al., 2024) are specifically designed for image classification processing and are not suitable for classifying table data after feature extraction. Therefore, based on the analysis and classification of chlorophyll fluorescence in tomato seedlings, this article compares several commonly used classification models for data analysis, BPNN (Qiao et al., 2019; Dong et al., 2020), NB (Cen et al., 2016), K-NN (Cen et al., 2016), SVM (Cen et al., 2017; Lu and Lu, 2021), with BiLSTM and DBO-BiLSTM models for cold injury classification and recognition, achieving the evaluation and comparison of classification models (**Table 9**).

**Table 9:** Evaluation of models for tomato cold damage classification and identification.

Algorithm model	Evaluation %			
	Accuracy	Precision	Recall	F1-measure
BPNN	82.22	91.67	78.57	84.62
NB	72.72	74.05	78.34	73.58
K-NN	77.28	73.41	75.99	73.54
SVM	89.10	89.10	89.50	89.30
BiLSTM	86.36	89.41	86.67	87.12
DBO-BiLSTM	95.45	96.43	95.83	95.80

As shown in **Table 9**, the Accuracy, Precision, Recall, and F1 mean of BPNN, NB, K-NN, SVM, BiLSTM, and DBO-BiLSTM classification models were calculated based on the most relevant features to cold damage, aiming to evaluate the effectiveness of multiple models in identifying chilling injury in the table. The top three models for Accuracy, Recall, and F1 mean were SVM, BiLSTM, and DBO-BiLSTM, while the top three models for Precision were BPNN, BiLSTM, and DBO-BiLSTM. In the experiment of chilling injury classification and prediction, the DBO-BiLSTM model optimized by DBO parameters improved the evaluation parameters Accuracy, Precision, Recall, and F1 mean by 9.09, 7.02, 9.16, and 8.68, respectively, compared to the original BiLSTM model. The evaluation parameters Accuracy, Precision, Recall, and F1 mean were 6.35, 7.33, 6.33, and 6.5 higher, respectively, than the SVM classification model. Obviously, the DBO-BiLSTM gives a better prediction in the experiment. The accuracy, precision, recall, and F1-measure of the training and test sets of the BiLSTM optimized by the DBO algorithm are all improved by at least 7% and all exceed 95%. Cen et al. (2016) implemented supervised classifications using NB, SVM, and KNN for two-class (normal vs. chilling) and three-class (normal, lightly chilling, severely chilling) categorizations based on spectral and image analysis at selected two-band ratios. For two-class recognition, SFS with NB, SVM, and KNN achieved accuracies of 97.6%, 100%, and 98.8%, respectively, with SVM outperforming. For three-class recognition, MIFS with the same classifiers yielded accuracies >80%, specifically 81.0%, 88.1%, and 83.3%, with SVM again the most accurate. However, for tomato leaves’ chilling injury into four categories, SRCC-SVM yielded 89.1% accuracy, likely due to chlorophyll fluorescence image characteristics and feature selection methods. Qiao et al. (2019) reported BPNN recognition accuracy increasing with blueberry decay grade, reaching 92.5% for severe decay. Dong et al. (2020) used color descriptors and fluorescence parameters with BPNN for chilling injury classification, achieving 90% accuracy. However, in this study, the recognition rate of BPNN was only 82.22% when classified according to cold damage areas,potentially due to input data selection. Leveraging the time series nature of class features, the DBO-BiLSTM model significantly improved recognition accuracy by 13.23% over BPNN, indicating its potential for deep learning in plant stress identification.

In conclusion, it was feasible to classify tomato cold damage based on the proportion of the damaged area in chlorophyll fluorescence imaging. Although the recognition performance for categories 2 and 3 in the internal classification was significantly lower than for categories 1 and 4, this was likely due to the segmentation of the image set and data. However, this phenomenon is consistent with the findings reported by Cen et al. (2016), wherein the application of the MIFS method in combination with SVM recognition was employed for the classification of three distinct types of cucumber cold damage. Additionally, after filtering features using SRCC, the conventional recognition methods like BPNN, NB, K-NN, and SVM showed average performance, and the evaluation parameters such as Accuracy, Precision, Recall, and F1 mean did not exceed 90%. However, once we enhanced the deep learning BiLSTM model using the Beetle Optimization (DBO) algorithm, the classification evaluation parameters accuracy, precision, recall, and F1 mean for tomato cold injury degree, based on chlorophyll fluorescence imaging, all surpassed 90%, indicating good results.

This study optimized the BiLSTM model leveraging the DBO algorithm, significantly advancing the accuracy of tomato cold injury classification via chlorophyll fluorescence imaging, comprehensively surpassing 90% of evaluation benchmarks, and bolstering agricultural intelligent monitoring and disease recognition automation. This achievement not only helps to provide timely warnings and reduce losses during crop growth, but also promotes the breeding of low-temperature tolerant tomato varieties, enhances crop stress resistance, and promotes sustainable agricultural development. Moreover, this method’s broad applicability extends to other crops and integrates multi-source data, enhancing disease identification accuracy and robustness, introducing novel concepts and tools for intelligent agricultural management.

Future endeavors will delve deeper into the integration of algorithm optimization and deep learning to optimize model efficiency and precision. Concurrently, data augmentation and transfer learning techniques will be harnessed to bolster the model’s generalization capacity and adaptability to dynamic environments. Additionally, interdisciplinary collaborations across plant physiology, computer science, and agricultural engineering will be fortified, deepening the understanding of the relationship between chlorophyll fluorescence and crop physiology, thereby underpinning precision management and efficient agricultural production.

## Data Availability

The raw data supporting the conclusions of this article will be made available by the authors, without undue reservation.
